# Effect of alternative temozolomide schedules on glioblastoma *O*^6^-methylguanine-DNA methyltransferase activity and survival

**DOI:** 10.1038/sj.bjc.6605792

**Published:** 2010-07-13

**Authors:** C G Robinson, J M Palomo, G Rahmathulla, M McGraw, J Donze, L Liu, M A Vogelbaum

**Affiliations:** 1 Department of Radiation Oncology, Washington University in St Louis, 4921 Parkview Place, St Louis, MO 63110, USA; 2 Brain Tumor and Neuro-Oncology Center and Department of Neurosurgery, Cleveland Clinic, 9500 Euclid Avenue/Desk ND40, Cleveland, OH 44195, USA; 3 Division of Hematology/Oncology, Case Western Reserve University, 11100 Euclid Avenue, Cleveland, OH 44195, USA

**Keywords:** temozolomide, glioblastoma, *O*^6^-methylguanine-DNA methyltransferase, xenograft

## Abstract

**Background:**

*O*^6^-methylguanine-DNA methyltransferase (MGMT) expression in glioblastoma correlates with temozolomide resistance. Dose-intense temozolomide schedules deplete MGMT activity in peripheral blood mononuclear cells; however, no published data exist evaluating the effect of temozolomide schedules on intracranial tumour MGMT activity.

**Methods:**

Human glioblastoma cells (GBM43) with an unmethylated *MGMT* promoter were implanted intracranially in immunodeficient rodents. Three weeks later, animals received temozolomide 200 mg m^−2^ for 5 days (schedule A, standard dose) or 100 mg m^−2^ for 21 days (schedule B, dose intense).

**Results:**

Tumour MGMT activity was depleted by day 6 in both treatment groups compared with baseline. *O*^6^-methylguanine-DNA methyltransferase activity returned to baseline by day 22 in the schedule A group, but remained suppressed in the schedule B group. By day 29, MGMT activity had returned to baseline in both groups. Mean tumour volume was significantly decreased compared with untreated controls with either schedule (*P*<0.01), although neither schedule was superior (*P*=0.60). Median survival was 64, 42, and 28 days for schedule A, schedule B, and no drug, respectively (*P*<0.001 A or B *vs* control, *P*=NS A *vs* B).

**Conclusions:**

Dose-intense temozolomide prolongs tumour MGMT activity depletion compared with standard dosing, however, survival was not improved in this model.

In 2005, Stupp *et al* published the results of a landmark trial (EORTC 22981/26981, NCIC CE. 3) showing a significant survival improvement in patients with newly diagnosed glioblastoma treated with radiotherapy and temozolomide (Temodar, SCH52365, NSC362856, Schering-Plough, Kennilworth, NJ, USA) *vs* radiotherapy alone. Temozolomide is an oral alkylating agent that exerts its cytotoxic effect through methylation of the *O*^6^ position of guanine. The *O*^6^-methylguanine lesion is recognised by the DNA mismatch repair (MMR) pathway, and cytotoxicity is thought to result from repeated failure of MMR to repair the lesion, which results in DNA double-strand breaks and apoptosis (Bignami *et al*, 2000; Roos *et al*, 2007). *O*^6^-methylguanine-DNA methyltransferase (MGMT) is the sole enzyme capable of repairing the *O*^6^-methylguanine adduct, and the level of MGMT in cells is directly correlated with alkylating agent cytotoxicity (Dumenco *et al*, 1989; Schold *et al*, 1989). In a correlative study using a subset of tumour samples from EORTC 22981/26981 by Hegi *et al* (2005), median survival was significantly improved in those patients treated with temozolomide in whom tumour *MGMT* was inactivated by hypermethylation of CpG islands in the promoter region. Although epigenetic inactivation of *MGMT* is present in approximately 40% of newly diagnosed glioblastoma, the remaining tumours have normal or even elevated levels of MGMT and are resistant to treatment with temozolomide (Esteller *et al*, 1999; Paz *et al*, 2004). Thus, depletion of MGMT activity in these tumours should result in enhanced sensitivity to temozolomide.

One approach to inhibiting MGMT activity involves the delivery of temozolomide using dose-intense schedules that generate sufficiently large numbers of *O*^6^-methylguanine adducts such that total cellular MGMT is depleted. If the schedule could be delivered in a manner such that the generation of *O*^6^-methylguanine lesions was to outstrip the rate of MGMT synthesis, cytotoxicity should be enhanced. Early preclinical and clinical studies revealed temozolomide to have significant schedule-dependent anti-tumour activity, with more frequent dosing schedules producing greater cytotoxicity than less frequent schedules (Stevens *et al*, 1987; Newlands *et al*, 1997). Standard dose temozolomide, as delivered for its original FDA-approved indication in the treatment of recurrent high-grade glioma, is dosed at 150–200 mg m^−2^ for 5 days every 28 days (Yung *et al*, 1999, 2000). This dosing schedule was also used after concurrent temozolomide and radiation in EORTC 22981/26981. A number of studies have since been undertaken to test the safety and efficacy of dose-intense schedules of temozolomide in patients with newly diagnosed and recurrent high-grade glioma. The most commonly used dose-intense schedules treat patients with temozolomide at 150 mg m^−2^ given 7 days on, 7 days off, or 100 mg m^−2^ given 21 days on, 7 days off, both of which deliver a dose intensity of approximately 2.1 relative to the standard dose regimen (Wick *et al*, 2004, 2007; Athanassiou *et al*, 2005; Caroli *et al*, 2007; Chinot *et al*, 2007; Neyns *et al*, 2008). Although control rates using dose-intense temozolomide regimens seem to be improved compared with historical controls, Grade 3/4 lymphocytopenia rates ranging from 30 to 100% have been reported (Brock *et al*, 1998; Wick and Weller, 2005; Neyns *et al*, 2008).

To test the hypothesis that dose-intense treatment schedules of temozolomide might improve overall survival for patients with newly diagnosed glioblastoma, the Radiation Therapy Oncology Group (RTOG 0525) randomised over 1000 patients treated with surgical resection and external beam radiation therapy (60 Gy in 30fx) with concurrent temozolomide (75 mg m^−2^ daily during radiation) to adjuvant temozolomide on a (1) standard (200 mg m^−2^ for 5 days every 28 days) or (2) dose-intense schedule (100 mg m^−2^ for 21 days every 28 days). Enrollment has completed, and preliminary results are anticipated shortly.

Overall, our understanding of the kinetics of MGMT activity depletion by alkylating agents in normal tissues and tumour are limited. In a seminal phase I study by Tolcher *et al* (2003), dose-intense schedules of temozolomide were found to reliably deplete MGMT activity in peripheral blood mononuclear cells (PBMCs). To date, however, there are no studies evaluating the effect of dose-intense temozolomide schedules on MGMT activity in brain tumours. Therefore, we sought to characterise the effect of the clinically relevant standard and dose-intense temozolomide arms from RTOG 0525 on intracranial tumour MGMT activity and survival using an orthotopic xenograft model.

## Materials and Methods

### Glioblastoma model

GBM43 cells were generously provided by Jann Sarkaria (Department of Radiation Oncology, Mayo Clinic). GBM43 was originally derived from a 69-year-old man who underwent resection of a left temporal glioblastoma, and has been maintained as a part of a human GBM xenograft panel as previously described (Giannini *et al*, 2005; Sarkaria *et al*, 2006). GBM43 cells have been previously characterised as having an unmethylated *MGMT* promoter, and express functional MGMT protein (Kitange *et al*, 2009).

### Orthotopic xenograft model

All experiments were performed on a protocol approved by the Cleveland Clinic Institutional Animal Care and Use Committee (IACUC no. 08517). Animals were fed a standard rodent diet, and maintained in a pathogen-free environment. GBM43 cells were grown in short-term cell culture (7–14 days) at 37 °C with 5% CO_2_ in DMEM supplemented with 10% fetal bovine serum, and 1% antibiotic/antimycotic (Invitrogen, Carlsbad, CA, USA). Immediately before inoculation, cells were harvested by trypsinisation and suspended in PBS to a concentration of 1 × 10^6^ cells *μ*l^–1^. GBM43 cells were inoculated into the right basal ganglia of anaesthetised athymic nude mice (*nu/nu*, Charles River, Wilmington, MA, USA) or rats (*RNu*, Charles River) using a stereotactic frame (David Kopf Instruments, Tujunga, CA, USA). Mice were inoculated with 3 × 10^6^ GBM43 cells in a total volume of 3 *μ*l, and rats were inoculated with 5 × 10^6^ GBM43 cells in a total volume of 5 *μ*l.

### Temozolomide dosing schedules

Animals treated with the standard dose schedule (schedule A) received intraperitoneal temozolomide at a dose of 200 mg m^−2^ for 5 days, and those treated with the dose-intense schedule (schedule B) received temozolomide at a dose of 100 mg m^−2^ for 21 days. Body surface area (BSA) dosing was converted to mg kg^–1^ dosing using the BSA normalisation method (Reagan-Shaw *et al*, 2008). Using a *K*_*m*_ factor of 3 for mice, schedule A was given at 66.6 mg kg^−1^ for 5 days and schedule B was given at 33.3 mg kg^−1^ for 21 days. Using a *K*_*m*_ factor of 6 for rats, schedule A was given at 33.3 mg kg^−1^ for 5 days and schedule B was given at 16.7 mg kg^−1^ for 21 days. Reagent-grade temozolomide was obtained from OChem, Inc. (Des Plaines, IL, USA), and dissolved in PBS with 10% DMSO immediately before injection.

### Effect of temozolomide dosing on tumour MGMT activity

Three weeks after inoculation with GBM43, animals were randomised to treatment with temozolomide on schedule A or schedule B. At serial time points after the initiation of treatment, 2–3 animals per treatment group were killed and intracranial tumour was removed. Tumour was snap frozen in liquid nitrogen, and stored at −80 °C for subsequent MGMT activity analysis. Intracranial tumour was extracted from three animals on day 1 of the experiment for assessment of pretreatment tumour MGMT activity. In the rat cohort, intracranial tumour was extracted from two randomly selected animals in each treatment group on days 3, 6, 10, 15, and 22 after the initiation of treatment. In the mouse cohort, intracranial tumour was extracted from three randomly selected animals in each treatment group on days 6, 22, and 29 after the initiation of treatment. MGMT activity in frozen tumour samples was measured as removal of [^3^H]methyl adduct from the *O*^6^ position of guanine in DNA by incubating tissue extracts with excess [^3^H]methyl-DNA substrate as previously described (Gerson *et al*, 1986; Spiro *et al*, 1999). MGMT activity is reported as fmol O^6^ mG per *μ*g DNA.

### Effect of temozolomide dosing on tumour volume

Three weeks after inoculation with GBM43, mice were randomised to treatment with no drug, or temozolomide on schedule A or schedule B. On day 0, five untreated control mice were killed and the whole brain was removed. On day 22, all remaining mice were killed and the whole brain was removed. Whole brains were fixed in 10% paraformaldehyde for at least 3 h, and then washed three times for 10 min in PBS. After fixation, they were immersed in 10% sucrose in PBS overnight and then 20% sucrose in PBS until the sample reached the bottom of the tube. They were then frozen in OCT (Tissue-Tek – Sakura – OCT Compound, Sakura Finetek USA, Inc., Torrance, CA, USA) and 10 *μ*m cryosections were made (Leica CM3050 S cryostat, Leica Microsystems, GmbH, Wetzlar, Germany) and mounted on glass slides.

Slides were stained with hematoxylin and eosin and mounted using Cytoseal TM (Stephens Scientific, Wayne, NJ, USA) mounting medium. A whole tissue section on a slide was imaged with a Leica DM5000B microscope (Leica Microsystems) equipped with a Prior Motorised Stage and Linearly Encoded Controller (Prior Scientific, Inc., Rockland, MA, USA) and Retiga SRV Cooled CCD camera with Liquid Crystal tunable RGB filter (QImaging, Surrey, BC Canada). Through an automated ‘tiled-mosaic’ process, this microscope allows the acquisition of an image of an entire tissue sample with a 5 × magnification. Tumours were measured using ImagePro software (Version 6.1.0.346, Media Cybernetics, Bethesda, MD, USA), and tumour volumes were calculated using a modified ellipsoidal formula: 1/2(length × width^2^) where length is the greatest longitudinal diameter and width is the greatest transverse diameter. The effect of temozolomide treatment on tumour volume was evaluated using the two-tailed Students' *t*-test.

### Effect of temozolomide dosing on survival

Three weeks after inoculation with GBM43, 30 mice were randomised to treatment with no drug (*n*=10), schedule A (*n*=10), or schedule B (*n*=10). Mice were examined daily, and were killed if they developed abnormal behaviour, evidence of pain or distress, or >20% weight loss. Survival was evaluated using the Kaplan–Meier method, and was calculated from the first day of treatment. Survival between temozolomide treatment schedules was compared using the log-rank test.

## Results

### Effect of temozolomide dosing on tumour MGMT activity

**Figure 1:**
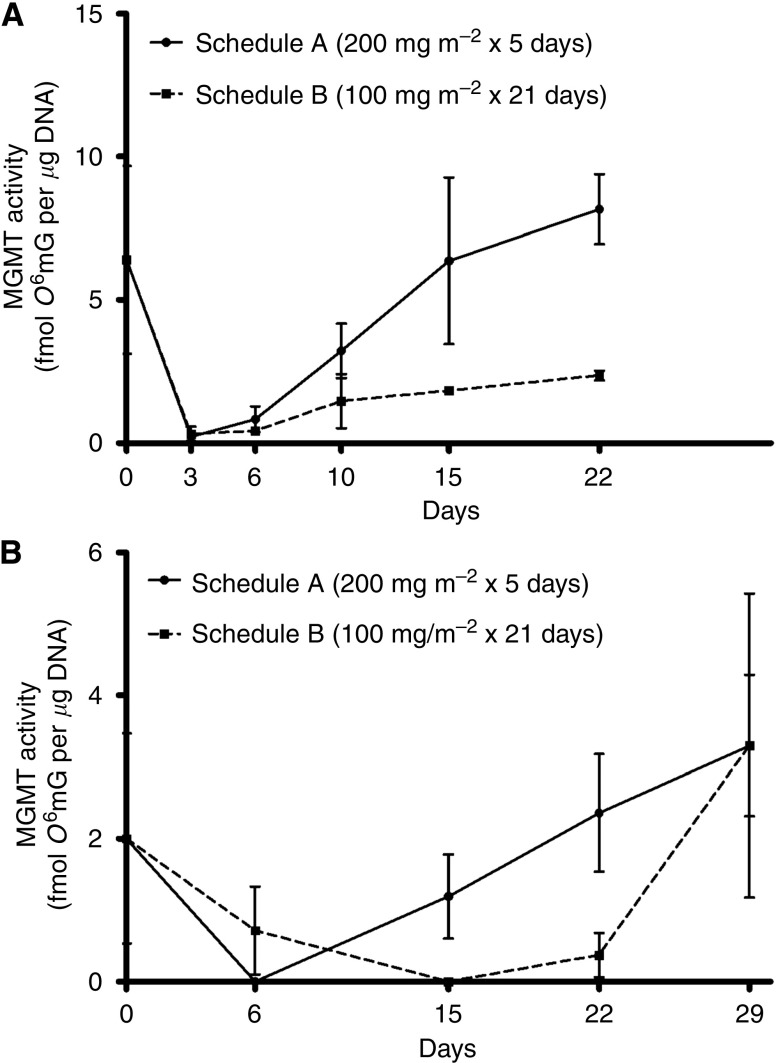
Plots of mean (s.d.) MGMT activity in orthotopic human GBM extracted from (**A**) *RNu* rats, during and after treatment with temozolomide on schedule A or B, on days 0 (pretreatment), 3, 6, 10, 15, and 22; and (**B**) *nu/nu* mice, during and after treatment with temozolomide on schedule A or B, on days 0 (pretreatment), 6, 15, 22, and 29.

*RNu* rats inoculated orthotopically with GBM43 tumour were treated with temozolomide using a standard dose (schedule A, 200 mg m^−2^ for 5 days) or dose-intense (schedule B, 100 mg m^−2^ for 21 days) schedule. Mean MGMT activity from the extracted brain tumours is plotted in Figure 1A, and tabulated in Table 1. Three days after the initiation of treatment, both schedules depleted tumour MGMT activity to <5% of pretreatment levels. On day 6, MGMT activity in tumour remained suppressed in both groups. By day 10 MGMT activity returned to 50% of pretreatment levels in the Schedule A group, but remained at <25% of pretreatment levels in the schedule B group. In the schedule A group, tumour MGMT activity returned to pretreatment levels by day 15, and remained elevated on day 22. In contrast, tumour MGMT activity in the schedule B group remained suppressed to 30–40% of pretreatment levels on day 15 and 22. It is noteworthy that while the dose-intense schedule effectively suppressed MGMT activity during the course of treatment, activity seemed to rebound somewhat during days 10–22 compared with the maximum suppression noted on days 3 and 6.

We next sought to confirm the above results, and additionally examine whether MGMT activity in tumour treated on the dose-intense schedule might return to pretreatment levels in the week after completing treatment, as it did with the standard dose schedule. *Nu/nu* mice were inoculated with GBM43 tumour and treated exactly as described in the first experiment, with the exception of the choice of time points for evaluation of tumour MGMT activity. Mean MGMT activity from the extracted brain tumours is plotted in Figure 1B, and tabulated in Table 1. As with the first experiment, by day 6 MGMT activity in tumour was effectively suppressed in both groups, and rapidly returned to pretreatment levels after the completion of treatment in the schedule A group. In contrast to the results of the first experiment, tumour MGMT activity on schedule B remained near undetectable on day 15 and 22, and did not show an appreciable rebound. However, by day 29, 1 week after the completion of treatment on schedule B, tumour MGMT activity had returned to pretreatment levels. The combined results from both experiments indicate that treatment with a dose-intense temozolomide schedule results in more prolonged suppression of MGMT activity than a standard dose schedule, but activity quickly returns after cessation of daily treatment.

### Effect of temozolomide dosing on tumour volume

**Figure 2:**
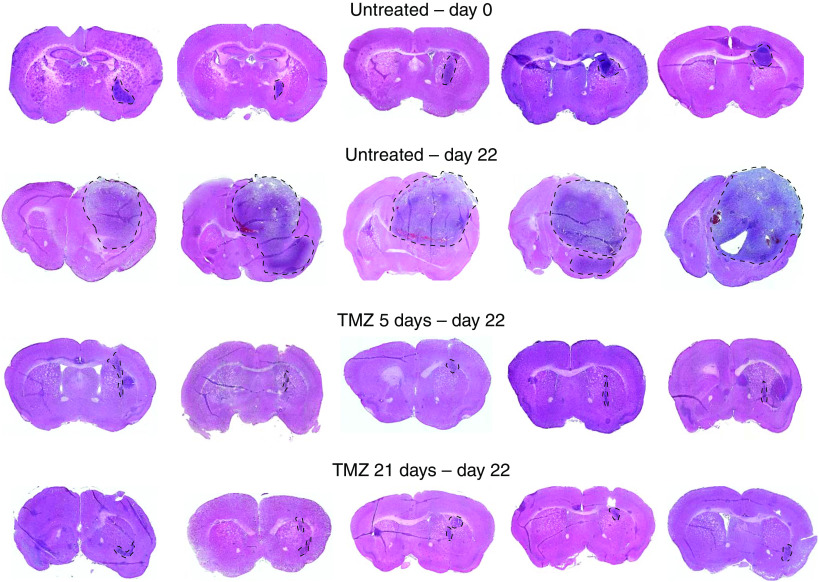
Photomicrographs of representative 10-micron sections showing the largest cross-sectional tumour for each mouse inoculated with human GBM. Tumour is outlined in representative sections. Calculated tumour volumes are shown in Table 2.

For the next set of experiments, we sought to determine whether the more prolonged depletion of MGMT activity using the dose-intense temozolomide schedule translated into improved tumour control and survival. To first determine the effect of the dose-intense temozolomide schedule on tumour volume, mice bearing GBM43 tumours were randomly assigned 21 days after tumour inoculation to immediate killing or treatment with no drug, Schedule A, or schedule B (five mice per treatment group). Mice were killed and brains harvested on day 0 (no drug) or day 22 (no drug, schedule A and B) after the start of temozolomide treatment. Whole mount sections were made through the region of the tumour and the section with the largest cross-sectional diameter tumour was evaluated for tumour volume measurements. The representative sections are shown in Figure 2 and the tumour volume calculations are shown in Table 2. When compared with the untreated group at day 22, both the schedule A and schedule B tumours were smaller (*P*<0.01). However, no difference was observed between the tumour volumes after treatment on schedule A or B (*P*=0.60).

### Effect of temozolomide dosing on survival

**Figure 3:**
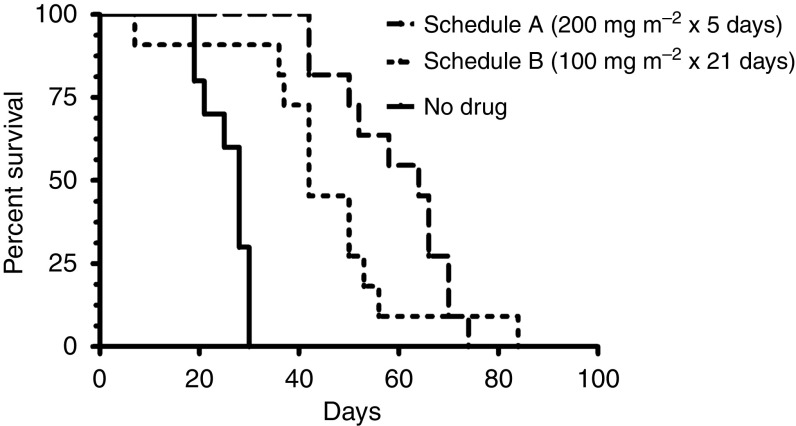
Kaplan–Meier survival curve of mice inoculated with orthotopic human GBM and treated with temozolomide on schedule A, B, or no drug.

To determine the effect of the dose-intense temozolomide schedule on survival, mice inoculated with GBM43 tumour were randomly assigned to treatment with no drug, schedule A or schedule B. Survival for the three treatment arms is plotted in Figure 3. The median survival was 64, 42, and 28 days for schedule A, schedule B, and no drug, respectively. Median survival for mice treated with schedule A or B was significantly greater than no drug (A *vs* no drug, HR 4.60, *P*<0.0001; B *vs* no drug, HR 3.69, *P*=0.0001). Median survival for schedule A *vs* B was not significantly different (HR 1.52, *P*=0.124).

## Discussion

Multiple studies have now validated the clinical importance of MGMT expression as a marker for resistance to temozolomide in the treatment of malignant glioma (Jaeckle *et al*, 1998; Esteller *et al*, 1999, 2000; Hegi *et al*, 2005; Chinot *et al*, 2007). Less than 40% of patients with newly diagnosed glioblastoma have inhibited expression of MGMT because of hypermethylation of the *MGMT* promoter, and 2-year survival in this group when treated with temozolomide approached 50% on the EORTC 22981/26981 study. The remaining patients typically have normal, or even increased tumour MGMT expression, and 2-year survival in this group is dismal, at <15% (Hegi *et al*, 2005). Although MMR and p53 mutation status also seem to have a role in the expression of temozolomide-induced cytotoxicity, MGMT is a clearly definable target with multiple potential methods to deplete its activity (Liu *et al*, 1996; Hermisson *et al*, 2006). Owing to the mechanism by which MGMT repairs *O*^6^-methylguanine adducts and is subsequently degraded, the use of alternative dosing regimens is an appealing method to enhance temzolomide mediated tumour cytotoxicity. Although multiple preliminary lines of evidence suggest enhanced clinical response rates with dose-intense regimens, there are no published studies to date documenting the effect of these temozolomide schedules on MGMT activity in brain tumour. As such a study would be very difficult to perform in humans, we attempted to measure the effects of standard and dose-intense temozolomide schedules on human glioblastoma MGMT activity using an orthotopic xenograft model. Furthermore, we sought to characterise the effect of dose-intense temozolomide dosing might have on survival. We surmised that the results of these studies would be informative for understanding the cause underlying the success or failure of RTOG 0525.

In this study, immunocompromised rodents were inoculated with a human GBM line previously characterised as having an unmethylated *MGMT* promoter and expressing functional MGMT protein (Kitange *et al*, 2009). This particular line was taken from an established xenograft panel serially propogated in *Nu/Nu* mouse flanks, which has been characterised as maintaining the key histopathologic features of glioblastoma including necrosis, invasion, and nuclear pleomorphism (Sarkaria *et al*, 2008). After treatment with temozolomide on either standard dose (schedule A, 200 mg m^−2^ for 5 days) or dose-intense (schedule B, 100 mg m^−2^ for 21 days) schedules, MGMT activity in tumour was maximally depleted within 3–6 days after initiation of therapy. In the days after completion of 5 days of treatment on the standard dose arm, MGMT activity in tumour rapidly returned to pretreatment levels. In contrast, tumour extracted from animals treated with the dose-intense schedule was noted to have near complete suppression of MGMT activity throughout treatment. However, just 1 week after treatment with 21 days of temozolomide on the dose-intense schedule, tumour MGMT activity had returned to pretreatment levels. Thus, treatment with protracted temozolomide schedules does in fact lead to persistent suppression of MGMT activity, however, levels return rapidly to baseline with even a brief treatment break.

Our results are in keeping with those found by Tolcher *et al* (2003) in his study of MGMT levels in human PBMCs. Patients with solid malignancy refractory to standard therapy were enrolled onto consecutive phase I protocols evaluating two different dose-intense temozolomide regimens, and MGMT activity in PBMCs was measured. The first cohort received temozolomide given orally at escalating doses from 50 to 175 mg m^−2^ daily for 7 days every 14 days, and PBMCs were collected at baseline, day 8, and day 15 during the first cycle of treatment. MGMT activity in PBMCs was depleted to 28% of baseline on day 8, and recovered to 45% of baseline by day 15. MGMT activity on day 15 was significantly decreased compared with baseline (*P*<0.001), although did not seem to be significantly different between day 8 and day 15. The second cohort received oral temozolomide at escalating doses from 50 to 150 mg m^−2^ daily for 21 days every 28 days, and PBMCs were collected at baseline, day 15, and day 22. MGMT activity in PBMCs was significantly depleted compared with baseline on both day 15 (63% decrease, *P*<0.001) and day 22 (73% decrease, *P*<0.001). For minimally pretreated patients, maximum tolerated dose was 150 mg m^−2^ and 100 mg m^−2^ for the 7-day/14-day and 21-day/28-day regimens, respectively, and dose-limiting toxicity was secondary to thrombocytopenia and lymphopenia. Although no comparison was made with the standard dose regimen of 200 mg m^−2^ for 5 days every 28 days, this study clearly showed that dose-intense temozolomide schedules can effectively deplete MGMT activity in PBMCs.

Two previously reported studies have attempted to examine the effect of dose-intense temozolomide regimens on MGMT activity in tumour. In the first, Gander *et al* (1999) treated 24 patients (22 with metastatic melanoma, 2 with recurrent glioma) on a prospective trial evaluating escalating doses of temozolomide from 300 to 700 mg m^−2^ divided over 2 days. Patients were also treated with fotemustine 100 mg m^−2^ on day 2, given 4 h after the temozolomide dose. In a subset of six patients with accessible metastatic melanoma deposits, MGMT activity was assessed in PBMCs and tumour at baseline and 4 h after the initiation of treatment. After treatment with temozolomide, mean MGMT activity in tumour was depleted to 56% of pretreatment levels. It is noteworthy that in three of the six patients there was discordance between changes in MGMT activity in PBMCs and tumour – two patients had an increase in PBMC MGMT activity and a decrease in tumour levels, and one patient had a decrease in PBMC MGMT activity and an increase in tumour levels. In the second study, Spiro *et al* (2001) treated 22 patients with metastatic solid tumours on a phase I trial with either (1) a bolus dose of temozolomide 200 mg m^−2^ followed by escalating doses starting at 50 mg m^−2^ given every 12 h for 5 days every 28 days, or (2) temozolomide 200 mg m^−2^ given daily for 5 days every 28 days. MGMT activity was assessed in PBMCs at regular intervals in all patients, and in core biopsies of liver metastases at baseline and 2 h after the last dose of temozolomide in 15 patients. At 24 h, patients assigned to treatment with twice daily temozolomide had lower mean levels of MGMT activity in PBMCs compared with the once daily group (<10 *vs* 50% of baseline, *P*<0.0001). Baseline tumour MGMT activity varied significantly between patients, as well as between sections of a tumour biopsy in the same patient. After treatment, mean depletion in MGMT activity varied from 0 to 84% in patients treated with a cumulative dose of 1000 mg m^−2^, and varied from 20 to 78% in those who received 1010 mg m^−2^. Two patients received a cumulative dose of 1100 mg m^−2^, one of which showed 99.5% inactivation and the other showed no decrease at all. No clear correlation was noted between depletion of MGMT activity in PBMCs and tumour, nor was there a correlation between depletion of tumour MGMT activity and response rate. Although neither study used the more commonly used 7-day/14-day or 21-day/28-day regimens, nor was brain tumour specifically evaluated, both studies suggest that dose-intense temozolomide schedules can deplete MGMT activity in tumour. Common between the two studies was discordance in several patients between changes in MGMT activity in PBMCs and tumour.

Recently, Kitange *et al* (2009) attempted to further characterise changes in MGMT expression in several of the glioblastoma lines from the Mayo xenograft panel, which included assessment of the GBM43 line. After treatment of GBM43 cells in short-term culture with a single dose of 100 *μ*M temozolomide, they found a two-fold induction in MGMT expression 48 h after treatment, as quantified by both western blot and RT–PCR. The influence of temozolomide treatment on MGMT activity in tumour was then evaluated in mice with established flank GBM43 tumours. Mice were treated with temozolomide at 50 mg kg^−1^ daily for 5 days, and at serial time points (days 0, 1, and 7 after treatment), animals were killed and MGMT activity was assessed in flank tumour. Compared with MGMT activity on day 0, relative activity by day 7 had increased 13-fold. The results of this analysis are not directly comparable to our own, however, as no assessment was made of intracranial tumour activity, and all measurements of MGMT expression and activity were made *after* treatment with temozolomide. Nonetheless, these results indicate that treatment with temozolomide leads to increased MGMT expression in the GBM43 line, at least in the week after treatment. In this study, MGMT activity in intracranial tumour samples taken 1–2 weeks after treatment was not clearly higher than pretreatment levels, although the trend is suggestive.

After showing that dose-intense temozolomide results in more prolonged depletion of intracranial tumour MGMT activity compared with a standard dose schedule, we next sought to determine whether treatment with the dose-intense schedule might improve tumour response, as defined by assessment of pre- and post-treatment tumour volumes and survival. In both sets of experiments, mice with orthotopically implanted GBM43 were randomly assigned to treatment with no drug, temozolomide 200 mg m^−2^ daily for 5 days, or temozolomide 100 mg m^−2^ daily for 21 days. Tumour volumes at the completion of therapy for mice treated with either temozolomide schedule were significantly smaller compared with no treatment, however, there was no appreciable difference in final tumour volume between the two treatment schedules. Similarly, median survival for mice treated with either temozolomide schedule was significantly improved compared with no treatment. Again, however, there was no statistically significant difference in median survival between the two treatment schedules. Kitange *et al* (2009) also evaluated survival in mice with orthotopically implanted GBM43 treated with temozolomide using (1) 50 mg kg^−1^ daily for 5 days per week over 2 weeks (500 mg kg^−1^ total dose), (2) 120 mg kg^−1^ once a week for 2 weeks (240 mg kg^−1^ total dose), and (3) 120 mg kg^−1^ daily for 5 days (600 mg kg^−1^ total dose). Relative to treatment with vehicle alone, survival was 1.3 times control in mice treated with either the daily 50 mg kg^−1^ or twice-weekly 120 mg kg^−1^ schedules, and 2.0 times control in mice treated with the daily 120 mg kg^−1^ schedule. The daily 120 mg kg^−1^ schedule resulted in significantly improved survival compared with the other two schedules (*P*<0.01). The investigators conclude that the more protracted regimens resulted in inferior tumour control as a result of the induction in MGMT expression after treatment with temozolomide. Whether this rationale entirely explains the lack of a survival benefit to treatment with dose-dense temozolomide in this study is not clear. In fact, in the current analysis, the dose-dense regimen effectively suppressed MGMT activity in tumour, and thus any induction in expression of protein expression was presumably countered by inhibition of enzyme activity. However, although MGMT activity was reliably suppressed to levels below 20–30% of baseline, it is possible that even limited MGMT activity may be sufficient to repair the *O*^6^-methylguanine lesions generated by temozolomide. Thus, complete inhibition may be what is necessary to show an appreciable improvement in tumour control, and whether this is achievable with modifications in temozolomide scheduling alone remains to be determined.

In the recently completed RTOG 0525 phase III trial, patients with newly diagnosed glioblastoma underwent a maximal safe resection, followed by 6 weeks of temozolomide and radiation as delivered by the EORTC 22981/26981 regimen. Patients were randomised to treatment with 12 cycles of adjuvant temozolomide delivered using either a standard (200 mg m^−2^ daily for 5 days every 28 days) or dose-intense (100 mg m^−2^ daily for 21 days every 28 days) schedule. The design for the trial was based in large part on the work by Tolcher *et al* (2003), showing enhanced depletion of MGMT activity in the PBMCs of patients subjected to two different dose-intense temozolomide regimens.

Another unstated assumption of the RTOG 0525 trial is that modification of the final adjuvant temozolomide portion of treatment might be sufficient to affect overall survival. With the implementation of the EORTC 22981/26981 regimen as the new standard of care, temozolomide was simultaneously introduced concurrently with radiotherapy, and as part of a planned 6–12 cycles of continued adjuvant temozolomide using a standard dosing schedule. One recent publication has attempted to address the relative importance of the concurrent and adjuvant temozolomide portions of the EORTC 22981/26981 regimen. Sher *et al* (2008) retrospectively analysed the outcomes of 43 patients with glioblastoma treated at a single institution with surgical resection and involved field radiotherapy. In total, 21 patients received adjuvant temozolomide alone after radiotherapy, and 22 received temozolomide concurrent with radiotherapy, followed by additional adjuvant temozolomide. At a median follow-up of 33.7 months, the hazard ratio for survival trended towards significance in favour of treatment with concurrent and adjuvant temozolomide over adjuvant temozolomide alone (HR=0.51, *P*=0.08).

This study is the first to methodically assess changes in intracranial glioblastoma MGMT based on modifications in temozolomide dosing. Using the clinically relevant schedules from the RTOG 0525 trial, we determined that a dose-intense temozolomide schedule is more effective than a standard dose schedule at depleting MGMT activity in human glioblastoma using an orthotopic xenograft model. In spite of the improved MGMT activity depletion, there was no apparent survival benefit to dose-intense temozolomide when the same model was challenged with the two schedules, in this single treatment cycle model of glioblastoma. Regardless of the absence of a survival benefit, the tumour MGMT activity results lend validity to the basic premise of the RTOG 0525 trial and other ongoing trials using dose-intense temozolomide regimens in the treatment of newly diagnosed or refractory glioblastoma. Results from the recently completed RTOG 0525 trial are anxiously awaited.

## Figures and Tables

**Table 1:** Mean MGMT activity in brain tumours extracted from *RNu* rats and *nu/nu* mice treated with temozolomide on schedule A or B

**Day**	**Mean MGMT activity in brain tumour (fmol MGMT *μ*g^−1^ DNA)**			
	**Rat**			
	**No drug**	**s.d.**		
0	6.40	3.27		
	**Schedule A**	**s.d.**	**Schedule B**	**s.d.**
3	0.22	0.13	0.32	0.25
6	0.83	0.45	0.42	0.11
10	3.22	0.95	1.46	0.95
15	6.37	2.91	1.83	0.13
22	8.17	1.21	2.36	0.17
	**Mouse**			
	**No drug**	**s.d.**		
0	2.19	1.47		
	**Schedule A**	**s.d.**	**Schedule B**	**s.d.**
6	0^a^	0^a^	0.715	0.62
15	1.19	0.59	0^a^	0^a^
22	2.36	0.82	0.37	0.31
29	3.30	2.12	3.30	0.99

Abbreviation: MGMT, *O*^6^-methylguanine-DNA methyltransferase.

a A value of ‘0’ means that there was no detectable MGMT activity in any of the samples.

**Table 2:** Volumes of orthotopic GBM43 tumours in *nu/nu* mice untreated (treatment day 0 or 22) or treated with temozolomide on schedule A or B (treatment day 22)

	**Tumour volumes (mm^3^)**			
***N***	**No treatment day 0**	**No treatment day 22**	**Schedule A day 22**	**Schedule B day 22**
1	0.1094	23.4256	0.0026	0.0161
2	0.1228	12.1377	0.0030	0.0041
3	0.0674	10.5504	0.0019	0.0122
4	0.0367	9.1602	0.0282	0.0095
5	0.1427	3.9681	0.0055	0.0144
Average	0.0958	11.8484	0.0082	0.0112
s.d.	0.0430	7.1611	0.0112	0.0047
*P*-value	—	—	*P*<0.01 (Schedule A *vs* no treatment)	*P*<0.01 (Schedule B *vs* no treatment)
			*P*=0.60 (Schedule A *vs* schedule B)	
